# Soil–plant interactions and metal uptake efficiency of native species in phosphate mining-affected environments

**DOI:** 10.1186/s12870-026-08832-z

**Published:** 2026-05-11

**Authors:** Samah Ramadan, Muhammad Rizwan, Siham M. Al-Balawi, Maha M. Elshamy

**Affiliations:** 1 Botany Department, Faculty of Science, Mansoura University, Mansoura, 35516 Egypt; 2 Department of Environmental Sciences, Government College University Faisalabad, Faisalabad, 38000 Pakistan; 3 Department of Biology, Faculty of Science, University of Tabuk, P.O. Box: 741, Tabuk, 71491 Saudi Arabia

**Keywords:** Phytoremediation, Trace metal contamination, Phosphate mining soils, Native wild plant species, Metal bioaccumulation, Soil–plant interactions

## Abstract

**Background:**

Wild plant species serve as significant models for evaluating the effects of anthropogenic activities on terrestrial ecosystems. In recent years, phytoremediation has garnered significant attention as a sustainable approach to remediate metal-contaminated soils, due to its ability to maintain soil structure while facilitating potential metal recovery. This study assessed the phytoremediation capabilities of seven indigenous wild plant species in soils impacted by phosphate mining operations.

**Methods:**

Metal concentrations in soil and plant samples were measured by atomic spectroscopy. Ten biologically distinct rhizosphere soil samples were collected for each of the seven wild plant species; each biological replicate was made up of pooled material from five individual plants. The effectiveness of phytoremediation was evaluated by computing the Biological Accumulation Coefficient (BAC), Bioaccumulation Factor (BF), Element Accumulation Index (EAI), and Translocation Factor (TF) for selected trace metals.

**Results and discussion:**

Metal bioavailability was significantly associated with soil pH and organic matter content. The concentration of soil macronutrients was low. The bioconcentration factor exceeded one for most tested elements. Mn and Zn in *Bidens pilosa* and *Conyza bonariensis* were the sole exceptions. All metals except Mn had biological accumulation coefficients greater than 1. TF values suggested variable translocation potential among the studied plant species.

**Conclusion:**

The results indicate that the examined native plant species can tolerate elevated metal concentrations and sequester them, thereby endorsing their potential application in the remediation of phosphate-mining-affected metal-contaminated soils.

**Supplementary Information:**

The online version contains supplementary material available at 10.1186/s12870-026-08832-z.

## Introduction

Phosphate ore is one of Egypt’s primary economic deposits. The Nile Valley near Idfu, the Red Sea between Safaga and Quesir, and the western desert between the El-Karga and El-Dakhla Oases are the three main areas in Egypt where the minefields are located. An estimated 3 billion tons of phosphate are present in Egypt’s deposits [1]. Over the past three decades, increasing awareness has focused on minimizing the environmental impacts of mining activities.

Mining processes and associated industrial processes have a significant impact on ecosystems in the vicinity. Although many phosphate mines are in arid regions of the Western Desert, the extraction, washing, loading, and transportation of ore may influence nearby environments, including soil quality, vegetation, groundwater, and natural habitats [2]. Soil in mining areas has altered physical and chemical properties that can limit microbial activity, plant growth, and the soil-forming process [3]. In addition to concentrations of potentially toxic metals, other limiting factors, such as soil compaction, nutrient depletion, and a lack of topsoil, may further limit vegetation establishment [4]. Despite these harsh conditions, several native plant species flourish and often develop adaptive traits that enable them to tolerate elevated metal concentrations, including *Erica andevalensis*, *Erica australis*, *Alhagi maurorum* and *Zygophyllum coccineum*. As a result, these plants have been extensively investigated for use in phytoremediation of heavy-metal-rich mine tailings [5–8]. Plants growing in metal-rich soils employ various physiological and biochemical mechanisms to cope with metal stress, including restricted metal uptake and transport, detoxification through sequestration in vacuoles, and activation of antioxidant defense systems [9]. Exposure to elevated metal concentrations can also induce oxidative stress, disrupt nutrient balance, and alter plant metabolic processes, while species-specific responses may enhance tolerance under contaminated conditions [10, 11].

Understanding how soil properties interact with plant metal uptake is crucial for assessing phytoremediation potential and minimizing the transfer of metals into ecological and food chains. Soil factors such as pH, electrical conductivity, and organic matter can affect metal movement and availability, which, in turn, affects how much metal plants accumulate. Native plants that thrive in metalliferous soil can help stabilize contaminated areas, reduce erosion, and limit the spread of metalliferous particles by wind or water [12].

Plants growing in metal-contaminated environments are often grouped according to their tolerance to metals. These groups include excluders, which limit metal uptake or restrict metal movement to aerial parts, and accumulators, which actively absorb and transport metals to the shoots [13, 14]. In phytoremediation research, these strategies are often assessed using indices like the Biological Accumulation Coefficient (BAC), Bioaccumulation Factor (BF), Translocation Factor (TF), and Element Accumulation Index (EAI). These indices help identify plant species that may be suitable for phytoextraction, which involves improved metal movement to shoots, or phytostabilization, in which metals are retained in the roots, in contaminated ecosystems [15, 16].

Many studies and reports in geology, geochemistry, and radiology have investigated the relationships between phosphate mining and other metals [17–19]. Some researchers have also examined the plant cover, anatomy, and phytochemical characteristics in mining sites [20–22]. Additionally, several plants outside Egypt have been reported to accumulate metals associated with phosphate mining activities [23–26]. However, there is little literature and limited data available about soil/plant interaction/or heavy metal accumulation by native wild plant species in arid phosphate mining regions in Egypt.

Several native medicinal plants grow in the Abu-Tartur phosphate mining district, an extremely arid region in Egypt’s Western Desert. Among them, five species belong to the Leguminosae family: *Astragalus vogelii* (Webb) Bornm., an annual sprawling herb used as animal feed with a medicinal root [27]; *Trigonella hamosa* L., an annual herb whose seeds are known to have steroid saponin-type compounds believed to lower lipid levels and regulate glucose [28]; *Melilotus indicus* (L.) All., a yellow flowering, highly sought after forage crop for its flowers as a source of nectar for honeybees, insecticide use and soil enhancer [29]; *Senna italica* Mill., a perennial woody shrub traditionally used in the treatment of various diseases (leaves, pods and hence also seeds are included in this category) [30]; and *Sesbania sesban* (L.) Merr., a non-deciduous legume tree that can tolerate saline and nutrient-deficient acidic soils and is often utilized as a nitrogen-fixing plant for improving nutrient content in soils [31, 32]. Two additional species belong to the Asteraceae family: *Bidens pilosa* Linn. (annual herb high in saponins, flavonoids, steroids and alkaloids) [33, 34]; *Conyza bonariensis* (L.) Cronquist (annual herb forms rosettes recognized for its antibacterial properties) [35].

In this conceptual framework, phosphate mining activities may alter soil properties, thereby influencing metal availability and plant uptake. Native plant species growing naturally in these environments may exhibit different tolerance strategies, including accumulation or exclusion. Evaluating metal concentrations in soils and plant tissues, together with accumulation indices, provides an approach to assess phytoremediation potential in mining-affected ecosystems. Therefore, this study aimed to assess the ability of selected native wild plant species growing in the Abu-Tartur phosphate mining area in the Western Desert, Egypt, to accumulate metals associated with mining activities. The phytoremediation potential of these species was determined by measuring their metal accumulation (Mn, Zn, Cu, Pb, Cd, and Ni) in soil and plant tissues and calculating accumulation indices. Plants with BAC/BF values greater than 1.0 and TF values greater than 1.0 were considered to have potential for phytoextraction.

In contrast, species with greater root retention and TF < 1 were interpreted as having phytostabilization potential. This study provides an initial screening of native plants adapted to mining-affected soils and contributes to understanding soil–plant interactions in arid phosphate mining ecosystems. This study provides new insights into soil–plant interactions in arid phosphate mining ecosystems and identifies native plant species with potential applications in ecological restoration of mining-affected lands.

## Materials and methods

### Study area

**Fig. 1:**
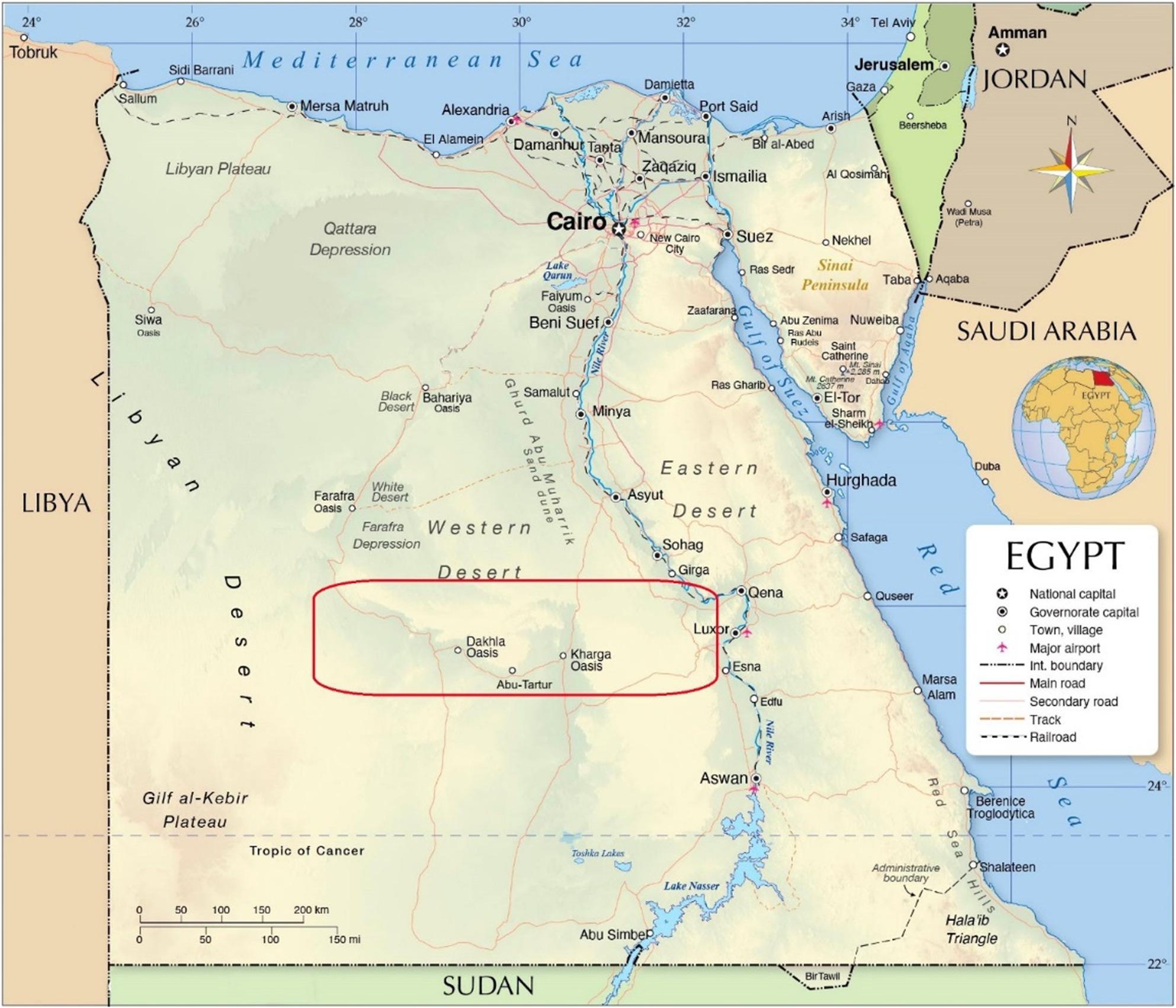
Map of phosphate Mining region of Abu-Tartur, Western Desert, Egypt. (according to El- Shamy 2016)

The phosphate deposit in Abu Tartur is among the Middle East’s most significant phosphate mining areas, with proven reserves of 1 billion tons and 200 million tons. Situated in the Egyptian Western Desert, the mining area is approximately 10 km away from the main road connecting the El-Kharga and El-Dakhla oases and 60 km away from El-Kharga city (Fig. 1). The Abu Tartur phosphate mining region is situated at a longitude of 30° 5’ 34” east and a latitude of 25° 22’ 50” north [20].

The study area is located in subtropical deserts. The climate is characterized by hot summers and mild winters. In January, the average temperature remains relatively stable between 12 °C and 4 °C; however, in July, it increases to approximately 39 °C. The region under consideration is characterized by a hyper-arid climate, with an annual precipitation of 0.1 mm and an average monthly evaporation rate of 414.66 mm [21].

### Soil and plant collection

In February 2021, all plants samples (*Sesbania sesban* (Authentication number: Sse3671), *Trigonella hamosa* (Authentication number: Th3994), *Conyza bonariensis* (Authentication number: Cbo1460), *Senna italica* (Authentication number: Si3572), *Melilotus indicus* (Authentication number: Mi2511), *Bidens pilosa* (Authentication number: Bp1198), and *Astragalus vogelii* (Authentication number: Av 925) were collected from their natural habitat around the Abu-Tartur phosphate mine site (30° 5’ 34” east and 25° 22’ 50” north) and deposited into the Herbarium of Faculty of Science, Mansoura University. The classification and characteristics (scientific name, common name, order, family, life span, life form, floristic category) of the seven native medicinal plants are illustrated in Table 1.

**Table 1:** Common name, order, family, life span, life form, floristic category of the studied species

No.	Taxon	Common name	Order	Family	Life-span	Life-form	Floristic category
1	*Astragalus vogelii* (Webb) Bornm.	Quarn	Fabales	Fabaceae	Ann	Th	SA
2	*Bidens Pilosa* L.	Black-jack	Asterales	Asteraceae	Ann	Th	PAN
3	*Conyza bonariensis* (L.) Cronquist	Hairy fleabane	Asterales	Asteraceae	Ann	Th	ME
4	*Melilotus indicus* (L.) All	Sweet-clover	Fabales	Fabaceae	Ann	Th	ME + IR-TR + SA
5	*Senna italica* Mill.	Senegal senna	Fabales	Fabaceae	Per	G	SA + SZ
6	*Sesbania sesban* (L.) Merr.	Egyptian rattle pod	Fabales	Fabaceae	Per	Ph	SA
7	*Trigonella hamosa* L.	Branched fenugreek	Fabales	Fabaceae	Ann	Th	ME + ES

Life span: *Ann* annual, *Per* perennial, Life form: *Th* Therophytes, *G* Geophytes, *Ph* Phanerophytes, Floristic category: *SA* Saharo -Arabian, *PAN* Pantropical, *ME* Mediterranean, *IR-TR* Irano-Turanian, *SZ* Sudano-Zambzian, *ES* Euro-Siberian

Soil and plant samples were collected simultaneously to assess soil–plant relationships. For each species, rhizosphere soil samples were collected at a depth of 0–20 cm after removing surface debris. A total of ten biological replicates (*n* = 10 per species) were obtained. Each replicate consisted of a composite sample formed by pooling rhizosphere soil from five individual plants of the same species growing within the same microhabitat, ensuring direct soil–plant association. The collected soil samples were evenly distributed onto paper sheets, air-dried, meticulously blended, filtered through a 2 mm screen to remove fine particles or debris, and subsequently enclosed in plastic bags for physical and chemical examinations [36].

For plant sampling, ten biological replicates per species were collected, each consisting of five randomly selected individuals pooled from the same microhabitat as their corresponding soil samples. Plant samples were thoroughly washed with deionized water to remove dust and surface contaminants, then separated into roots and shoots. The Plants were identified as described in Boulos [37] at the Mansoura University Botany Department, Faculty of Science, Herbarium. Both the shoots and roots of the studied plants, as well as their soil samples, were examined for several characteristics, as explained below. Plant species were selected based on their natural abundance and ecological dominance at the study site, substantial aboveground biomass, extensive rooting systems, and ability to survive under metal-stressed arid conditions. Medicinal relevance was considered secondary. Where available, previous reports indicating metal tolerance or accumulation capacity were also considered.

### Analysis of soil and plant samples

Soil organic carbon (OC) was determined according to the method of Combs and Nathan [38]. Soil pH and electrical conductivity (EC) were measured in soil–water suspensions using a digital pH meter (Lutron pH 206) and a conductivity meter (YSI Model 33), respectively [23]. Soil pH was determined in a 1:5 (w/v) soil–water suspension, whereas EC was measured in a 1:2.5 soil–water extract and expressed as µS cm⁻¹. Instruments were calibrated daily using standard buffer and conductivity solutions. All reported values are presented as mean ± standard deviation (SD) of biological replicates (*n* = 10 per species).

Soil samples were oven-dried at 72 °C to constant weight and digested using an acid mixture of HNO₃/HCl/HClO₄ (1:2:2, v/v/v) following Temminghoff and Houba [39]. After digestion, the samples were diluted with 0.2% HNO₃ and analyzed for macronutrients and micronutrients (Mg, K, Ca, S, Na, and Si) as well as trace elements (Zn, Fe, Cu, Mn, Mo, Cr, Se, Pb, B, Cd, Ni, and Ag).

Plant samples were dried at 72 °C for 48 h, separated into roots and shoots, and ground into a fine powder. The plant material was then digested according to the method described by Temminghoff and Houba [39]. Element concentrations in both soil and plant digests were determined using inductively coupled plasma–atomic emission spectrometry (ICP-AES; Ultima 2 JY, Horiba, Edison, NJ, USA). Total nitrogen (N) and phosphorus (P) in soil and plant samples were determined according to standard methods described by the American Public Health Association [40].

Quality assurance and quality control (QA/QC) procedures were strictly followed throughout the analysis. Instrument calibration was performed using multi-element standard solutions prepared from certified stock standards, and calibration curves showed high linearity (R² ≥ 0.999). Analytical precision was evaluated by triplicate analysis of selected samples, yielding relative standard deviation (RSD) values below 5%. Accuracy was assessed using spiked samples, with recovery rates ranging from 90 to 108%. Estimated limits of detection (LOD) for the analyzed elements were determined based on instrument sensitivity and are provided in Supplementary Table S1. All measured concentrations in soil and plant samples were above the respective detection limits.

### Biological accumulation coefficient, bioconcentration factor, translocation factor, and element accumulation index

Three factors were computed to assess the metal enrichment of the studied taxa [41]:

The concentration of metals in plant shoots divided by the concentration of metals in the soil around roots is known as the biological accumulation coefficient (BAC).

### BAC = [Metal] shoot / [Metal] soil

By dividing the metal content of roots by the metal content of the rhizosphere, one can determine the bioaccumulation factor (BF). This factor demonstrates the ability of roots to accumulate heavy metals.

### BF = [Metal] root / [Metal] soil

The shoot-to-root metal ratio transfer capacity is known as the translocation factor (TF).

### TF = [Metal] shoot / [Metal] root

The total effectiveness of metal accumulation in plants was evaluated by means of the element accumulation index (EAI) [42]:

### EAI = (1/n) Σ (C_i_ / C̄_i_)

where *Ci* is the concentration of element *i* in plant tissues, *C̄i* is the mean concentration of the same element across all studied species, and *n* is the total number of analyzed elements. EAI provides an integrated measure of overall metal accumulation performance across multiple elements.

### Data analysis

Statistical analyses were performed using R software (version 4.3.2; R Foundation for Statistical Computing, Vienna, Austria). Data were first tested for normality using the Shapiro–Wilk test and for homogeneity of variances using Levene’s test. Plant macronutrients (%) and micronutrients/trace metals (mg kg⁻¹) were analyzed separately.

Differences among plant species and tissues were evaluated using two-way analysis of variance (ANOVA), while differences among species were assessed using one-way ANOVA, followed by Tukey’s HSD post hoc test for pairwise comparisons of means. Different letters in the tables and figures indicate statistically significant differences among groups at *p* < 0.05. All statistical analyses were conducted using ten biological replicates per species (*n* = 10), and data are presented as mean ± standard deviation.

Each biological replicate represented one composite soil sample and its corresponding composite plant sample, with each composite formed by pooling material from five individual plants of the same species collected within the same microhabitat. Where technical replicates were performed during laboratory measurements, their mean value was calculated first and used as a single value for each biological replicate; technical replicates were not treated as independent observations in the statistical analyses.

Before principal component analysis (PCA), all variables were standardized to z-scores. PCA was performed using a combined dataset including metal concentrations in roots and shoots together with soil physicochemical properties, with biological replicates (*n* = 10 per species) used as observations. PCA was applied as an exploratory approach to visualize multivariate relationships among soil and plant variables.

Pearson correlation analysis was used to assess relationships among soil properties and elemental concentrations. Correlation matrices were visualized using heatmaps, and only statistically significant correlations were interpreted. No correction for multiple testing was applied, and this is acknowledged as a limitation of the study.

## Results

### Soil characteristics and element correlations

Soils from the phosphate mining region of the Western Desert were generally nutrient-poor, with particularly low levels of K, N, Mg, and Ca, consistent with the harsh conditions of this desert environment (Table 2). Soil physicochemical properties, including pH, EC, and organic matter content, varied significantly among plant habitats, indicating marked spatial heterogeneity within the mining area. Among the studied habitats, soil associated with *Astragalus vogelii* showed the highest pH and EC values and was also characterized by elevated concentrations of several elements, including K, Mn, Pb, Cd, Si, and Na. In contrast, soil associated with *Trigonella hamosa* was distinguished by comparatively higher Ca, Fe, and Cr concentrations. Other species showed element-specific enrichments, with *Melilotus indicus* associated with higher Se and Ag, *S. italica* with higher N, P, Mo, and B, *Bidens pilosa* with higher Mg, and *Conyza bonariensis* with higher S and Zn. Overall, most measured elements differed significantly among species, whereas Mg, N, Cu, Ni, and Pb showed no significant variation (Table 2).

**Table 2:** Chemical Characteristics of soil Samples associated with the studied species

Studied species		Astragalus vogalii	Sesbania sesban	Trigonella hamosa	Melilotus indicus	Senna italica	Bidens pilosa	Conyza bonariensis
pH		8.6 ± 0.25 ^b^	8.0 ± 0.28 ^ab^	7.9 ± 0.27 ^ab^	8.4 ± 0.29 ^ab^	7.4 ± 0.21 ^c^	7.5 ± 0.17 ^ab^	7.7 ± 0.22 ^ab^
EC ( µS cm⁻¹)		944,000 ± 21,800 ^a^	884,000 ± 25,520^ab^	771,000 ± 17,810^cd^	78,000 ± 22,520^bc^	668,000 ± 23,100^d^	673,000 ± 23,310^d^	707,000 ± 16,330^cd^
OC %		1.6 ± 0.03 ^bc^	1.4 ± 0.04 ^cde^	1.8 ± 0.04 ^a^	1.7 ± 0.06 ^ab^	1.5 ± 0.04 ^cd^	1.3 ± 0.03 ^e^	1.3 ± 0.03 ^de^
N	mg/Kg	44.9 ± 1.29 ^b^	44.0 ± 1.02 ^b^	42.8 ± 0.99 ^b^	42.6 ± 1.23 ^b^	46.9 ± 1.62 ^b^	44.7 ± 1.29 ^b^	43.1 ± 1.00 ^b^
P		14.3 ± 0.41 ^b^	16.8 ± 0.39 ^a^	17.00 ± 0.39 ^a^	16.9 ± 0.49 ^a^	17.3 ± 0.40 ^a^	17.0 ± 0.49 ^a^	16.2 ± 0.56 ^ab^
K		17.3 ± 4.99 ^a^	16.4 ± 3.78 ^a^	8.7 ± 2.01 ^b^	8.9 ± 2.57 ^b^	16.3 ± 5.64 ^a^	8.4 ± 2.41 ^b^	7.8 ± 1.81 ^b^
Ca		21.1 ± 0.61 ^c^	24.2 ± 0.56 ^bc^	24.9 ± 0.86 ^b^	22.5 ± 0.78 ^bc^	23.3 ± 0.68 ^bc^	23.3 ± 0.67 ^bc^	22.8 ± 0.79 ^bc^
Mg		14.1 ± 0.40 ^b^	13.2 ± 0.31 ^b^	14.8 ± 0.51 ^b^	14.8 ± 0.34 ^b^	13.4 ± 0.39 ^b^	14.8 ± 0.43 ^b^	14.8 ± 0.51 ^b^
S		9.8 ± 0.28 ^de^	9.9 ± 0.23 ^de^	18.1 ± 0.63 ^c^	5.7 ± 0.13 ^f^	8.4 ± 0.24 ^e^	11.9 ± 0.34 ^d^	37.6 ± 0.87 ^b^
Fe		3592.0 ± 0.01 ^d^	3910.0 ± 0.01 ^cd^	5324.0 ± 0.02 ^a^	5084.0 ± 0.01 ^ab^	4492.0 ± 0.01 ^b^	4928.0 ± 0.02 ^ab^	5052.0 ± 0.02 ^ab^
Mn		11992.0 ± 0.03 ^a^	5744.0 ± 0.02 ^c^	4760.0 ± 0.01 ^cd^	5564.0 ± 0.01 ^c^	4120.0 ± 0.01 ^d^	8272.0 ± 0.02 ^b^	4856.0 ± 0.01^cd^
Zn		448.0 ± 0.00 ^c^	384.0 ± 0.00 ^c^	488.0 ± 0.00 ^c^	688.0 ± 0.00 ^b^	124.0 ± 0.00 ^d^	376.0 ± 0.00 ^c^	1460.0 ± 0.00 ^a^
Cu		1358.8 ± 0.04 ^a^	1355.2 ± 0.03 ^a^	1362.8 ± 0.03 ^a^	1367.6 ± 0.05 ^a^	1371.6 ± 0.04 ^a^	1365.2 ± 0.05 ^a^	1361.6 ± 0.05 ^a^
Mo		1372.0 ± 0.03 ^b^	100.8 ± 0.00 ^d^	532.0 ± 0.01 ^c^	657.6 ± 0.02 ^c^	1830.4 ± 0.05 ^a^	100.0 ± 0.01 ^d^	1490.0 ± 0.05 ^b^
Se		656.8 ± 0.02 ^b^	440.8 ± 0.02 ^d^	639.6 ± 0.02 ^b^	803.6 ± 0.02 ^a^	439.2 ± 0.01 ^d^	538.8 ± 0.02 ^c^	676.0 ± 0.02 ^b^
B		922.8 ± 0.03 ^a^	882.0 ± 0.02 ^a^	572.0 ± 0.02 ^c^	584.8 ± 0.01 ^bc^	980.0 ± 0.03 ^a^	681.6 ± 0.02 ^b^	632.0 ± 0.02 ^bc^
Pb		942.4 ± 0.03 ^a^	928.4 ± 0.03 ^a^	915.6 ± 0.03 ^a^	922.0 ± 0.03 ^a^	933.2 ± 0.03 ^a^	898.4 ± 0.03 ^a^	919.6 ± 0.03 ^a^
Cr		23.6 ± 0.00 ^d^	148.4 ± 0.01^b^	202.4 ± 0.01^a^	8.4 ± 0.00^d^	94.4 ± 0.00 ^c^	159.2 ± 0.01^b^	181.6 ± 0.00 ^a^
Cd		2206.4 ± 0.06 ^a^	1581.6 ± 0.05 ^b^	1483.2 ± 0.04 ^b^	1563.6 ± 0.05^b^	1419.2 ± 0.05 ^b^	1834.4 ± 0.27 ^b^	1492.8 ± 0.16 ^b^
Ni		187.6 ± 0.01 ^a^	207.2 ± 0.01 ^a^	196.4 ± 0.01 ^a^	192.0 ± 0.01 ^a^	198.8 ± 0.01 ^a^	206.4 ± 0.01^a^	201.2 ± 0.01^a^
Ag		64.0 ± 0.00 ^d^	1302.4 ± 0.05 ^a^	782.0 ± 0.03 ^b^	1326.0 ± 0.03 ^a^	772.0 ± 0.03 ^b^	466.8 ± 0.01 ^c^	852.8 ± 0.02 ^b^
Si		226.6 ± 0.01 ^a^	78.4 ± 0.00 ^b^	56.8 ± 0.00 ^c^	58.8 ± 0.00 ^bc^	56.3 ± 0.00 ^c^	65.6 ± 0.00 ^bc^	63.2 ± 0.00 ^bc^
Na		1594.0 ± 0.05 ^a^	1577.0 ± 0.04 ^ab^	1396.4 ± 0.05 ^ab^	1406.4 ± 0.05 ^ab^	1486.0 ± 0.03 ^ab^	1419.6 ± 0.04 ^ab^	1387.6 ± 0.03 ^b^

*ns* non-significant

Results are presented as mean ± standard deviation (SD) based on 10 biological replicates per species. Different superscript letters indicate significant differences among means at *p* < 0.05

* *p* < 0.05, ** *p* < 0.01, *** *p* < 0.001

**Fig. 2:**
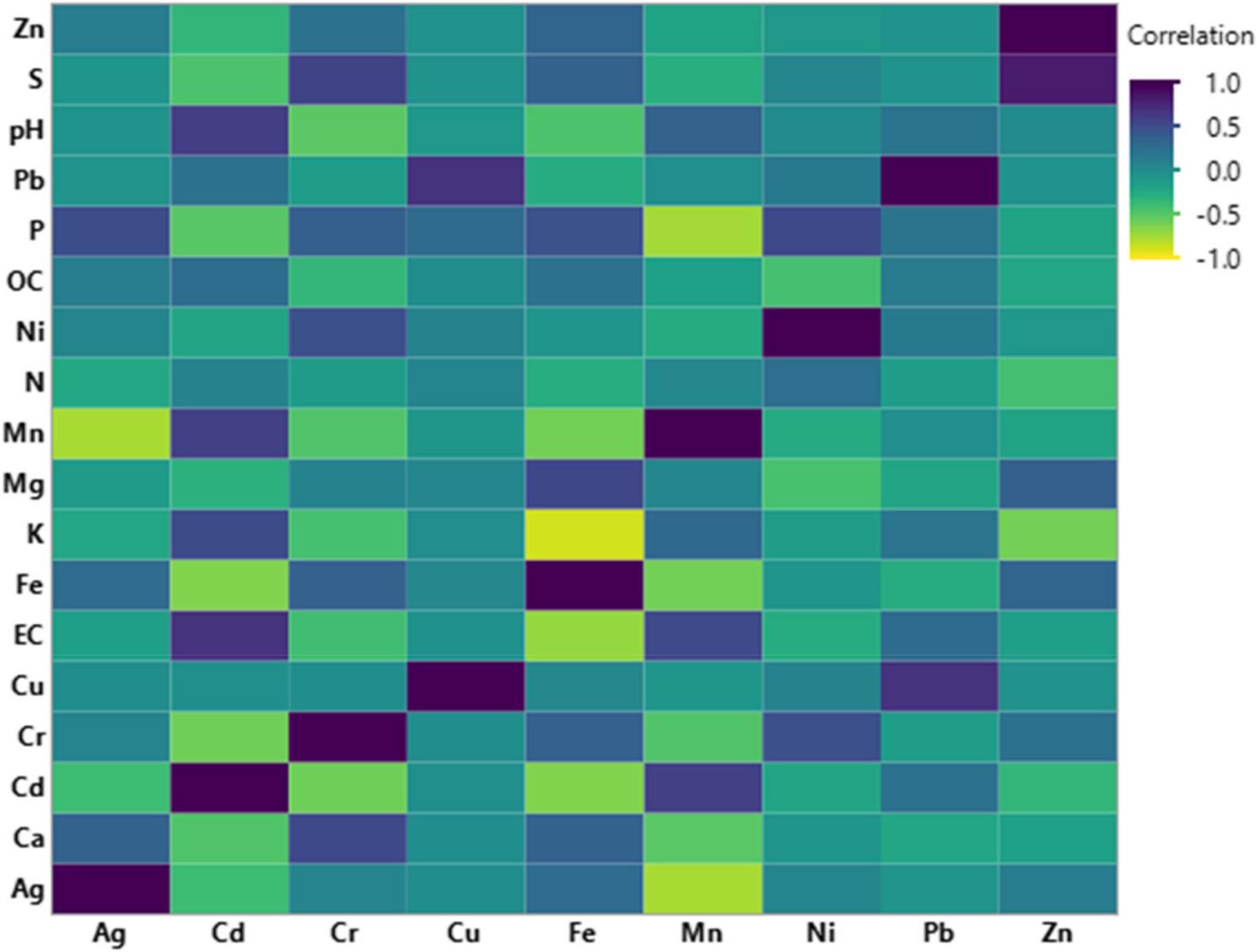
Heatmap showing Pearson correlation coefficients between soil properties (pH, EC, OC, N, P, S, Ca, Mg, K, Mn, Fe) and trace metals (Ag, Cd, Cr, Cu, Fe, Mn, Ni, Pb, Zn). Color intensity indicates correlation strength from −1 (yellow) to +1 (purple). Correlations were considered significant at P ≤ 0.05

The resulting heatmap presents Pearson correlation coefficients between the studied pH, EC, OC, macro-, micro- and trace elements in the analyzed soil samples, based on Pearson correlation (Fig. 2). Phosphorus had significantly positive correlations with Ag, Fe, Ni and Cr (*p* < 0.05), and significant negative correlations with Mn, Cd and Zn. Potassium was positively correlated with Cd, Mn, Pb, and Cu, while Fe, Zn, and Cr demonstrated negative correlations. Calcium was positively correlated with Cr, Ag, and Fe. Magnesium showed positive correlations with Fe, Zn, Cr and Cu, while sulfur was significantly positively correlated with Zn, Cr and Fe (Fig. 2).

Regarding soil physicochemical parameters (Fig. 2), pH showed negative correlations with Cr and Fe and positive associations with Cd, Mn, and Pb. Electrical conductivity (EC) showed positive correlations with Cd, Mn, and Pb but negative associations with Fe, Cr, Ni, and Ag. OC showed positive correlations with Cd, Fe, and Pb.

Among trace elements, Zn showed significant positive correlations with Fe, Cr, and Ag. Pb was positively correlated with Cu and Cd, and, for Ni, showed positive associations with Cr, Pb, and Ag. Mn showed positive correlations with Cd and Pb. In contrast, Fe showed positive correlations with Cr, Ag, and Zn (Fig. 2). Because multiple correlations were examined simultaneously, these relationships should be interpreted cautiously and represent exploratory associations rather than definitive interactions.

### Plant macro-and micronutrients and trace elements

Mining sites, plant species, and plant organs significantly affected the concentrations of all measured macroelements (N, P, K, Ca, Mg, and S) in the studied taxa (Table 3). Clear interspecific differences in macronutrient accumulation were observed. *Bidens pilosa* was characterized by comparatively higher N, P, and Ca concentrations, whereas *Sesbania sesban* showed greater accumulation of K and S. In contrast, *Astragalus vogelii* was distinguished by the highest Mg concentration (Table 3). These findings indicate marked species-specific differences in macronutrient uptake and allocation under mining-area conditions.

**Table 3:** Mean values of macronutrients in the shoots and roots of the study plant species collected from Abu-Tartur mining site., Western Desert, Egypt

Macroelements			*Astragalus vogelii*	*Sesbania* * sesban*	*Trigonella hamosa*	*Melilotus indicus*	*Senna italica*	*Bidens pilosa*	*Conyza* * bonariensis*
%	N	shoot	1.5±0.043^ab^	1.3±0.030^a^	1.4±0.033^a^	1.5±0.035^ab^	1.7±0.049^b^	2.3±0.081^c^	1.4±0.050^a^
		root	2.00±0.057^c^	1.3±0.044^a^	1.5±0.051^ab^	1.4±0.031^ab^	1.4±0.040^ab^	1.4±0.049^ab^	1.5±0.053^b^
	P	shoot	0.4±0.013^a^	0.4±0.010^a^	0.4±0.009^a^	0.4±0.009^a^	0.4±0.012^a^	0.5±0.010^a^	0.4±0.015^a^
		root	0.4±0.012^a^	0.4±0.010^a^	0.4±0.014^a^	0.4±0.015^a^	0.4±0.012^a^	0.5±0.011^a^	0.4±0.010^a^
	K	shoot	2.6±0.074^b^	3.0±0.068^c^	2.3±0.078^ab^	2.3±0.081^ab^	2.2±0.064^ab^	2.0±0.070^a^	2.1±0.049^a^
		root	2.8±0.082^b^	2.9±0.066^b^	2.2±0.050^a^	2.3±0.052^a^	2.4±0.068^a^	2.4±0.082^a^	2.0±0.047^a^
	Ca	shoot	1.4±0.040^a^	1.4±0.032^a^	1.4±0.049^a^	1.5±0.035^a^	2.1±0.059^b^	2.0±0.045^b^	1.5±0.051^a^
		root	1.3±0.036^b^	1.0±0.023^a^	1.00±0.022^a^	1.4±0.041^c^	1.2±0.035^b^	1.6±0.036^c^	1.6±0.036^c^
	Mg	shoot	1.0±0.028^d^	0.2±0.005^a^	0.4±0.013^c^	0.2±0.007^b^	0.2±0.005^a^	0.4±0.013^c^	0.2±0.004^ab^
		root	0.9±0.027^e^	0.1±0.003^b^	0.3±0.007^c^	0.0±0.002^a^	0.0±0.001^a^	0.4±0.009^d^	0.0±0.001^a^
	S	shoot	0.02±6.059^ab^	0.09±20.568^d^	0.03±7.157^c^	0.02±5.849^ab^	0.02±5.583^ab^	0.02±4.933^b^	0.02±5.719^a^
		root	0.03±9.529^c^	0.06 ±14.385^e^	0. ±5.646^b^	0.02 ±4.983^a^	0.06±21.997^e^	0.02±5.584^b^	0.04±9.947^d^

*ns* non-significant

Results are presented as mean ± standard deviation (SD) based on 10 biological replicates per species. Different superscript letters indicate significant differences among means at p < 0.05

* p < 0.05, ** p < 0.01, *** p < 0.001

Micro- and trace element concentrations were also significantly influenced by mining site, species identity, and plant organ (Tables 4 and 5). Significant interspecific variation was observed for Zn, Mn, Se, Mo, Ag, Cr, B, Na, and Si, whereas Cu, Pb, Fe, Ni, and Cd showed no significant variation among species in roots and shoots (Table 4). Element distribution also differed between plant organs. In general, shoots showed greater accumulation of Fe in most species, as well as Zn and Se in the majority of taxa, whereas roots tended to accumulate Ag and Na more strongly (Table 4).

**Table 4:** Heavy metal concentrations in shoots and roots of the studied species (mg Kg^− 1^)

	Astragalus vogelii		Sesbania sesban	Trigonella hamosa	Melilotus indicus	Senna italica	Bidens pilosa	Conyza bonariensis
Fe	Shoot	8097 ± 1.870^a^	7533 ± 2.175^a^	7688 ± 1.775^a^	7446 ± 1.720^a^	7806 ± 2.253^a^	7446 ± 2.150^a^	7868 ± 2.725^a^
	Root	7812 ± 2.255^a^	7818 ± 1.806^a^	7186 ± 2.489^a^	7384 ± 2.132^a^	7570 ± 1.748^a^	7415 ± 2.569^a^	7855 ± 1.814^a^
Zn	Shoot	3945 ± 1.367^e^	1639 ± 0.473^c^	2490 ± 0.575^d^	2296 ± 0.663^d^	1250 ± 0.289^b^	11 ± 0.004^a^	248 ± 0.072^a^
	Root	2598 ± 0.750^e^	1304 ± 0.452^d^	4420 ± 0.153^ab^	6790 ± 0.157^bc^	4100 ± 0.118^a^	9160 ± 0.212^c^	2835 ± 0.982^e^
Na	Shoot	3595 ± 1.038^a^	5137 ± 1.780^b^	3459 ± 0.999^a^	3461 ± 0.799^a^	3481 ± 1.005^a^	3510 ± 0.811^a^	3483 ± 1.005^a^
	Root	3642 ± 1.051^a^	5105 ± 1.768^b^	3502 ± 1.213^a^	3501 ± 1.213^a^	3513 ± 1.014^a^	3577 ± 0.826^a^	3587 ± 1.035^a^
Cu	Shoot	3415 ± 1.183^a^	3407 ± 0.984^a^	3395 ± 0.784^a^	3405 ± 1.180^a^	3419 ± 0.790^a^	3412 ± 0.985^a^	3422 ± 0.790^a^
	Root	3404 ± 0.983^a^	3418 ± 1.184^a^	3395 ± 0.784^a^	3420 ± 0.987^a^	3414 ± 1.183^a^	3408 ± 0.787^a^	3395 ± 0.980^a^
Mo	Shoot	2642 ± 0.610^ab^	3113 ± 0.899^de^	2747 ± 0.952^cd^	2348 ± 0.813^b^	3003 ± 0.867^cde^	1364 ± 0.473^a^	3348 ± 0.966^e^
	Root	752 ± 0.217^b^	1051 ± 0.364^c^	156 ± 0.036^a^	2351 ± 0.679^e^	1976 ± 0.456^d^	5238 ± 1.210^f^	860 ± 0.248^bc^
Cd	Shoot	2515 ± 0.727^a^	2519 ± 0.872^a^	2519 ± 0.872^a^	2518 ± 0.727^a^	2519 ± 0.582^a^	2517 ± 0.582^a^	2520 ± 0.727^a^
	Root	2514 ± 0.582^a^	2518 ± 0.727^a^	2518 ± 0.582^a^	2518 ± 0.582^a^	2518 ± 0.727^a^	2518 ± 0.872^a^	2520 ± 0.583^a^
Se	Shoot	2462 ± 0.569^f^	779 ± 0.270^a^	1703 ± 0.393^e^	953 ± 0.220^b^	1137 ± 0.328^c^	1042 ± 0.3016^bc^	1310 ± 0.303^d^
	Root	1622 ± 0.468^d^	767 ± 0.266^a^	1567 ± 0.543^d^	845 ± 0.242^ab^	1561 ± 0.543^d^	1275 ± 0.294^c^	1022 ± 0.295^b^
Pb	Shoot	2356 ± 0.543^a^	2294 ± 0.530^a^	230 ± 0.664^a^	2332 ± 0.808^a^	2313 ± 0.669^a^	2312 ± 0.533^a^	2340 ± 0.809^a^
	Root	2250 ± 0.651^a^	2297 ± 0.796^a^	2327 ± 0.537^a^	2290 ± 0.660^a^	2291 ± 0.792^a^	2324 ± 0.670^a^	2303 ± 0.532^a^
Mn	Shoot	1717 ± 0.397^d^	1797 ± 0.519^d^	1429 ± 0.330^c^	1412 ± 0.489^c^	895 ± 0.258^ab^	1054 ± 0.304^b^	756 ± 0.175^a^
	Root	1436 ± 0.415^bc^	1367 ± 0.316^b^	1596 ± 0.553^c^	1048 ± 0.303^a^	1575 ± 0.364^c^	1422 ± 0.410^bc^	1304 ± 0.452^b^
Ag	Shoot	1462 ± 0.506^cd^	1457 ± 0.505^cd^	1639 ± 0.379^d^	3939 ± 0.910^e^	1146 ± 0.265^b^	1234 ± 0.356^bc^	236 ± 0.055^a^
	Root	952 ± 0.275^b^	5733 ± 1.324^f^	704 ± 0.163^ab^	4712 ± 1.632^e^	3155 ± 0.911^d^	1892 ± 0.437^c^	333 ± 0.096^a^
B	Shoot	1380 ± 0.479^a^	1373 ± 0.475^a^	1418 ± 0.328^a^	1535 ± 0.531^a^	2050 ± 0.593^b^	1964 ± 0.455^b^	1472 ± 0.340^a^
	Root	1259 ± 0.364^b^	1002 ± 0.231^a^	960 ± 0.334^a^	1437 ± 0.415^c^	1218 ± 0.282^b^	1545 ± 0.445^c^	1570 ± 0.363^c^
Ni	Shoot	507 ± 0.146^a^	494 ± 0.171^a^	506 ± 0.146^a^	507 ± 0.117^a^	512 ± 0.177^a^	511 ± 0.118^a^	498 ± 0.144^a^
	Root	502 ± 0.145^a^	502 ± 0.174^a^	510 ± 0.177^a^	492 ± 0.170^a^	468 ± 0.135^a^	501 ± 0.116^a^	508 ± 0.176^a^
Cr	Shoot	137 ± 0.030^a^	219 ± 0.063^b^	205 ± 0.070^b^	107 ± 0.024^a^	342 ± 0.098^c^	216 ± 0.051^b^	427 ± 0.098^d^
	Root	125 ± 0.045^a^	143 ± 0.034^ab^	373 ± 0.109^d^	349 ± 0.121^d^	160 ± 0.057^ab^	225 ± 0.064^c^	175 ± 0.059^b^
Si	Shoot	478 ± 0.138^b^	1455 ± 0.336^c^	130 ± 0.030^a^	111 ± 0.032^a^	134 ± 0.031^a^	146 ± 0.034^a^	124 ± 0.029^a^
	Root	382 ± 0.087^b^	1490 ± 0.429^c^	134 ± 0.030^a^	135 ± 0.031^a^	132 ± 0.030^a^	117 ± 0.034^a^	124 ± 0.029^a^

*ns* non-significant

Results are presented as mean ± standard deviation (SD) based on 10 biological replicates per species. Different superscript letters indicate significant differences among means at *p* < 0.05

* *p* < 0.05, ** *p* < 0.01, *** *p* < 0.001

**Table 5:** Results of two-way ANOVA of heavy metals concentrations of the seven native plant species

Effect	df	*N*	*P*	k	Ca	Mg	S	Fe	Mn	Zn	Cu	Mo	Se	B	Pb	Cr	Cd	Ni	Ag	Si	Na
Species	6	34.92^***^	6.85^***^	39.75^***^	59.99^***^	1251.93^***^	656.27^***^	2.11^ns^	59.77^***^	454.69^***^	0.01^ns^	142.97^***^	5.02**	55.76^***^	0.04^ns^	136.24^***^	0.00^ns^	0.32^ns^	801.01^***^	2116.76***	55.32***
Tissue part	1	18.09^***^	0.50^ns^	3.035^ns^	216.27^***^	215.62^***^	151.43^***^	1.12^ns^	22.14^***^	151.71^***^	0.00^ns^	543.41^ns^	0.15***	202.08^ns^	0.43^***^	14.53^**^	0.00^ns^	0.83^ns^	560.81^***^	1.22^ns^	0.49^ns^
Species* Tissue part	6	40.6^***^	1.43^ns^	4.05**	29.07^***^	18.35^***^	235.24^***^	0.69^ns^	68.47^***^	387.84^***^	0.01^ns^	519.93^***^	0.38^ns^	27.10^***^	0.22^ns^	301.04^***^	0.00^ns^	0.74^ns^	301.74^***^	3.96**	0.06^ns^

*ns* non-significant

*P* value 0.05

*: *p* < 0.05, **: *p* < 0.01, ***: *p* < 0.001

Distinct species-specific accumulation patterns were also evident across the analyzed elements. *Astragalus vogelii* showed the highest concentrations of Zn, Fe, and Se, whereas *Sesbania sesban* was characterized by elevated Si, Mn, and Na. *Bidens pilosa* accumulated comparatively higher B, Mo, and Ni, while *Conyza bonariensis* showed greater Cr, Pb, and Cd concentrations. In addition, *Melilotus indicus* exhibited the highest Ag concentration, and *S. italica* showed the highest Cu concentration (Tables 4 and 5).

**Fig. 3:**
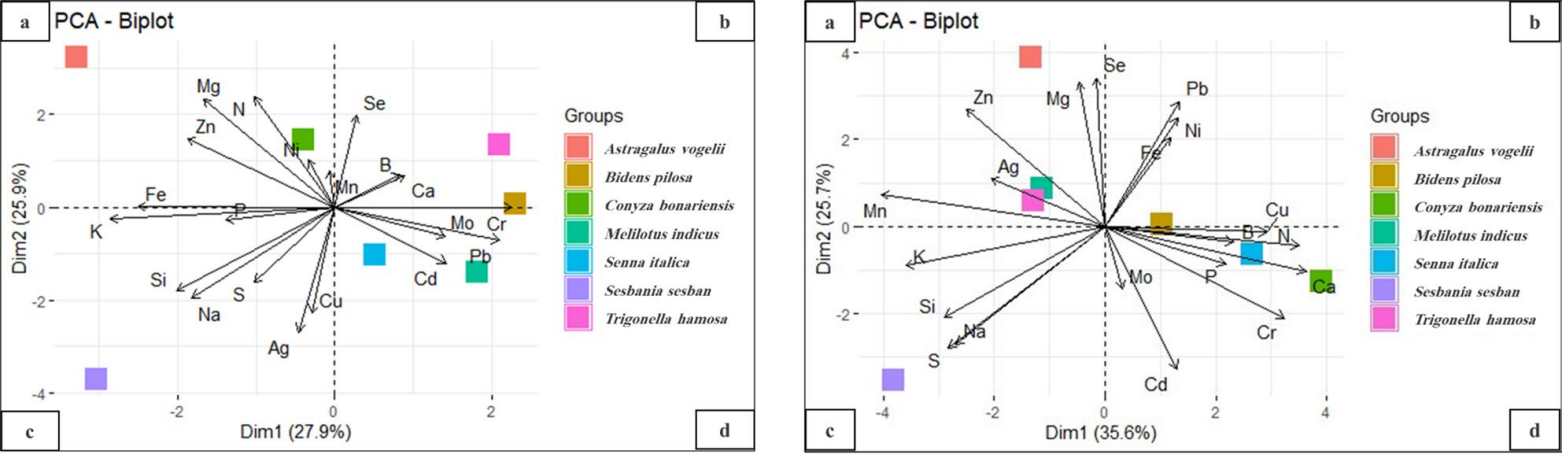
Principal component analysis (PCA) showing the relationships among macro- and microelement concentrations in the roots (Fig. 3**a**) and shoots (Fig. 3**b**) of the studied species on the component plane. **a** PCA-Ordination plot of roots of studied plants. **b** PCA-Ordination plot of shoots of studied plants

The Principal Components Analysis (PCA) method was applied to pattern recognition of all analytical variables, including all elements in the roots and shoots of the species under study (Fig. 3a and b). The PCA-biplot ordination of the studied species showed differences in element accumulation among these seven species and within tissues within each species.

Figure (3a) displays the roots’ accumulation ability along the two PCs components: PC1 (27.9%) and PC2 (25.9%). The biplot distributed the seven studied species into 3 groups: the first group, with N, Zn, Ni, and Mg variables associated with *(A) vogelii* and *C. bonariensis*. Along with the second component, the second group of Ca, Se, and Cr variables was associated with *(B) pilosa* and B for *T. hamosa*, and Pb, Mo, and Cd variables were associated with *M. indicus* and *S. italica*. The third group contained *S. sesban*, in which Fe, P, K, Na, Si, S, Cu and Ag differentiated.

Figure (3b) shows the shoot accumulation ability along the two PCs components: PC1 (35.6%) and PC2 (25.7%). The biplot also differentiated the seven studied species into 3 groups: the first group included *(A) vogelii*,* T. hamosa*, and *M. indicus*, which accumulated Mg, Se, Ag, Zn, and Mn in the same sector. The second group included *C. bonariensis*, *S. italica* and *(B) pilosa*, which were correlated with N, P, Ca, Cu, Cd, B, and Mo. In addition, the third group had only *S. sesban*, which was placed in the collection of variables that accumulated K, S, Si, and Na.

### Biological Accumulation Coefficient (BAC), Bioconcentration Factor (BF), Translocation Factor (TF) and Element Accumulation Index (EAI) of trace elements

BAC, BF, and TF were used as comparative indicators of metal uptake and internal translocation among the studied species. BAC or BF values > 1 indicate enhanced accumulation relative to soil concentrations, while TF > 1 reflects greater transfer of metals from roots to shoots. These indices are presented here to compare relative accumulation and translocation potential among species and tissues, rather than to infer definitive field-scale phytoextraction performance.

**Fig. 4:**
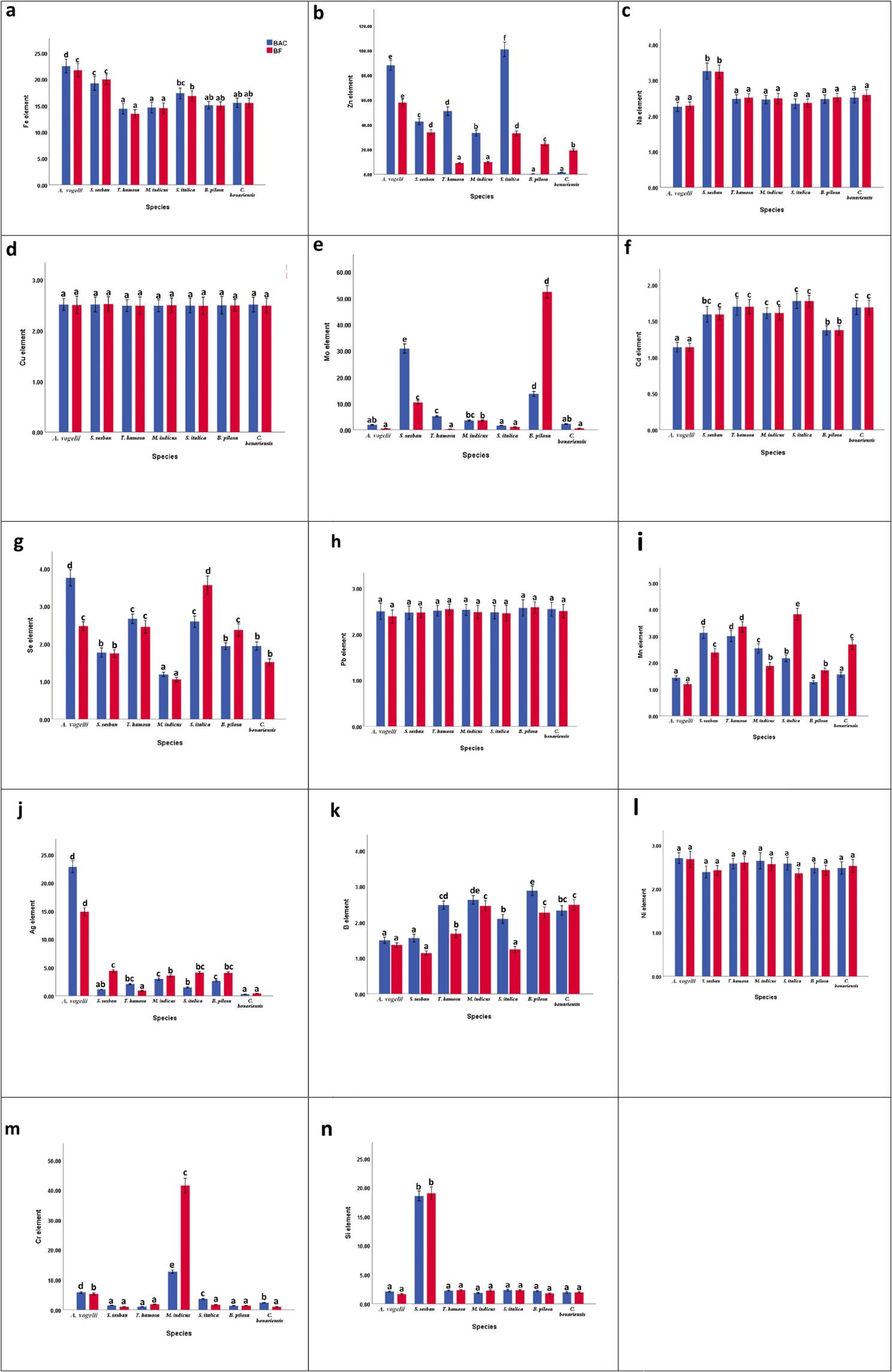
Mean values (± SD, based on 10 biological replicates) of translocation factors (TF) from soil to shoot tissues (blue bars) and bioaccumulation factors (BF) from soil to root tissues (red bars) for microelements in seven native plant species growing around the Abu-Tartur mining site, Western Desert, Egypt. Different superscript letters indicate significant differences among species according to one-way ANOVA followed by Tukey’s test (P < 0.05)

**Fig. 5:**
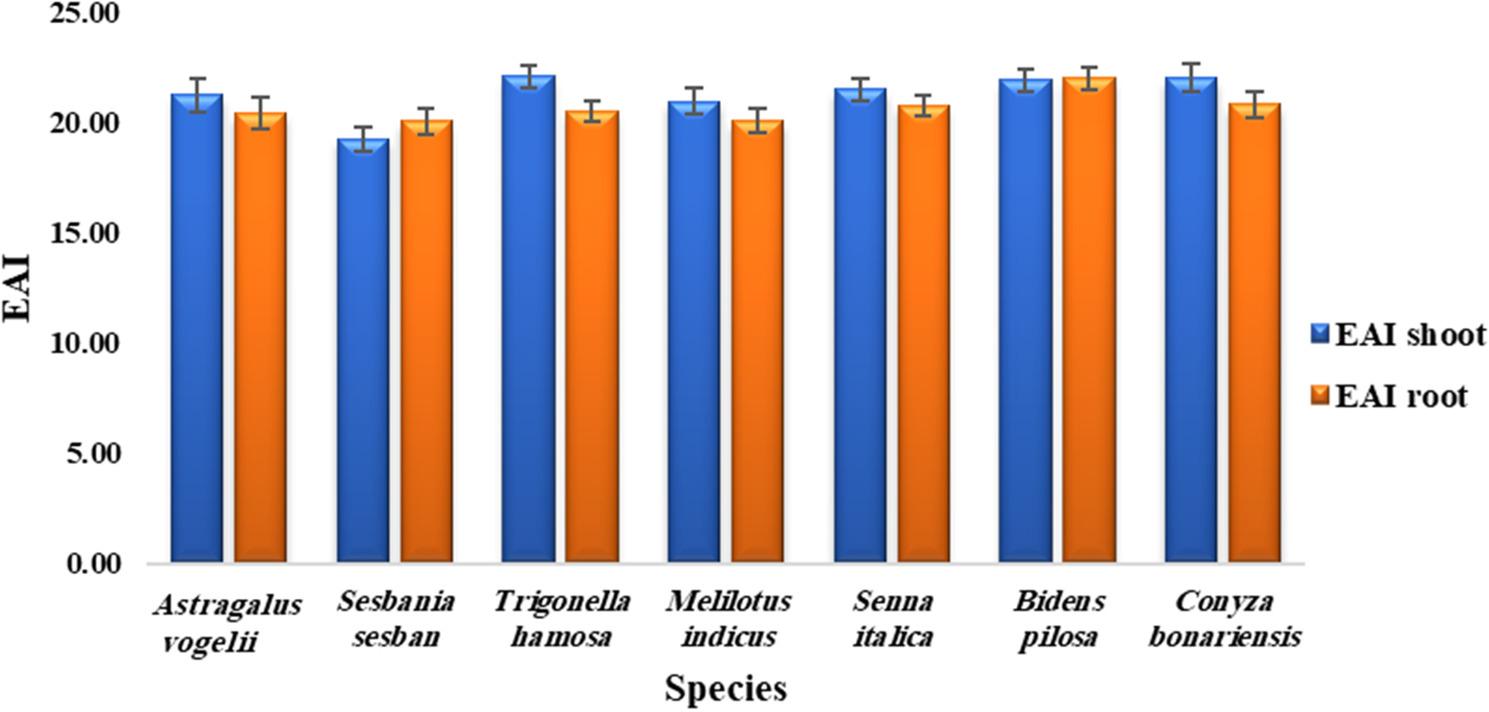
Element accumulation index (EAI) values in roots and shoots of the studied plant species growing around the Abu-Tartur phosphate mining site, Western Desert, Egypt. Values are presented as mean ± standard deviation (SD) based on 10 biological replicates

For the examined metals associated with the phosphate mining process, BAC, BF (Fig. 4), TF (Table 6), and EAI (Fig. 5) were computed. The findings demonstrated that the BACs of all heavy elements were greater than unity, excluding Mn in all species and Zn (0.03 and 0.17) in *B*. *pilosa* and *C. bonariensis*, respectively (Fig. 4). All tested species had BFs greater than one for all elements except Mn.

**Table 6:** Translocation factors (TFs) of microelements in the studied native plant species growing around the Abu-Tartur mining site, Western Desert, Egypt

	*Astragalus vogelii*		*Sesbania sesban*	*Trigonella hamosa*	*Melilotus indicus*	*Senna italica*	*Bidens pilosa*	*Conyza bonariensis*
TF	Fe	1.04 ± 0.036	0.96 ± 0.033	1.07 ± 0.031	1.07 ± 0.031	1.03 ± 0.036	1.00 ± 0.029	1.00 ± 0.023
	Mn	1.04 ± 0.030	0.96 ± 0.033	1.07 ± 0.031	1.01 ± 0.029	1.03 ± 0.024	1.00 ± 0.023	1.00 ± 0.035
	Zn	1.52 ± 0.044	1.26 ± 0.044	5.63 ± 0.130	3.38 ± 0.117	3.05 ± 0.106	0.01 ± 0.000	0.09 ± 0.003
	Cu	1.00 ± 0.029	0.99 ± 0.035	1.00 ± 0.023	0.99 ± 0.034	1.00 ± 0.035	1.00 ± 0.035	1.01 ± 0.029
	Mo	3.51 ± 0.081	2.96 ± 0.085	17.61 ± 0.610	0.99 ± 0.023	1.52 ± 0.044	0.26 ± 0.009	3.89 ± 0.113
	Se	1.52 ± 0.053	1.02 ± 0.035	1.09 ± 0.031	1.13 ± 0.039	0.73 ± 0.017	0.82 ± 0.024	1.28 ± 0.037
	B	1.10 ± 0.038	1.37 ± 0.040	1.48 ± 0.051	1.07 ± 0.031	1.68 ± 0.039	1.27 ± 0.044	0.94 ± 0.027
	Pb	1.37 ± 0.169	0.50 ± 0.248	1.88 ± 0.454	0.90 ± 0.067	0.58 ± 0.216	0.77 ± 0.115	0.81 ± 0.105
	Cr	1.10 ± 0.038	1.53 ± 0.044	0.55 ± 0.019	0.31 ± 0.009	2.14 ± 0.074	0.96 ± 0.022	2.44 ± 0.070
	Cd	1.00 ± 0.035	1.00 ± 0.029	1.00 ± 0.035	1.00 ± 0.029	1.00 ± 0.035	1.00 ± 0.023	1.00 ± 0.023
	Ni	1.01 ± 0.023	0.98 ± 0.028	0.99 ± 0.034	1.03 ± 0.030	1.09 ± 0.025	1.02 ± 0.029	0.98 ± 0.034
	Ag	1.54 ± 0.045	0.25 ± 0.008	2.33 ± 0.054	0.84 ± 0.024	0.36 ± 0.010	0.65 ± 0.019	0.73 ± 0.018
	Si	1.25 ± 0.043	0.98 ± 0.034	0.97 ± 0.028	0.82 ± 0.029	1.02 ± 0.029	1.25 ± 0.029	1.00 ± 0.029
	Na	0.99 ± 0.028	1.01 ± 0.023	0.99 ± 0.029	0.99 ± 0.034	0.99 ± 0.029	0.98 ± 0.023	0.97 ± 0.028

Results are presented as mean ± standard deviation (SD) based on 10 biological replicates per species

The TF varied between elements and species, and the BAC and BF for every heavy metal in the taxa under study were greater than unity. In general, the TFs for Fe, Ni, Cu, Cd, B, Mn, Si, Se and Na (excluding *A. vogelii*) and Zn (except *M. indicus*) were less than one for all roots of the investigated plants; however, they had a high percentage of BAC. TFs greater than unity for Mo, Zn, Ag and Se in *(A) vogelii*; Cr and Mo in *S. sesban*; Mo, Ag and Zn in *T. hamosa*; Cr, Mo and Zn in *S. italica*; Si and B in *(B) pilosa*; Cr and Mo in *(C) bonariensis* and Zn in *M. indicus*. indicating higher shoot translocation potential. Nevertheless, each species exhibited heavy metal selectivity, and the translocation potential was not consistent with the accumulation potential (Fig. 4).

In relation to the element accumulation index (EAI), all plant species’ root values were not significantly different from their shoot values (Fig. 5). *S. sesban* (19.28) had the lowest EAI among the species, while *T. hamosa* (22.15) and *C. bonariensis* (22.03) had the highest EAI. Furthermore, *M. indicus* (20.12) had the lowermost EAI of roots, and *B. pilosa* (22.04) had the uppermost.

## Discussion

The Abu-Tartur mining area is a long-established phosphate extraction site where soil disturbance and potential trace-metal enrichment create conditions relevant to phytoremediation assessment [43–46]. Although phosphate-bearing formations are naturally enriched with several essential elements, mining activities can alter soil structure, nutrient balance, and geochemical dynamics, thereby affecting plant establishment and metal behavior in the rhizosphere [47, 48].

In this study, soils exhibited slightly to moderately alkaline pH levels (7.4–8.6), which typically reduces the solubility of heavy metals through precipitation and adsorption [49]. However, total metal concentrations in soil do not accurately reflect bioavailable fractions, making plant tissue concentrations a better indicator of metal mobility and ecological risk [50]. Higher pH can affect metal distribution within soil pools, but results were based on total metal measurements rather than bioavailability [51]. Despite the alkaline conditions that usually limit metal availability, the species studied showed effective metal uptake, indicating that plant-specific traits significantly influence remediation potential. Variations in root accumulation and root-to-shoot translocation among species reveal different remediation strategies, with some plants focusing on phytostabilization while others prefer translocation [52]. Metal concentrations in soils and plants of phosphorite-rich desert ecosystems are strongly controlled by soil properties, plant traits, and the geochemical background created by phosphate mining [53–59]. Some native species can tolerate or accumulate high levels of metals, highlighting their value for soil stabilization, ecosystem restoration, and the reduction of mining-related environmental impacts [60].

Based on the heatmap correlation results, soil nitrogen and potassium showed the same trend: they positively correlated with Cd and Mn, and both negatively associated with Fe. N and K are essential nutrients for plants and are crucial for their growth and development. Yang et al. [61] reported that an increased amount of N, regardless of its form, enhances Cd uptake, translocation, and accumulation in plants, and nitrate promotes Cd uptake more than any other N form. Moreover, N is regarded as the most effective element that participates in the regulation of Cd uptake and accumulation compared to other elements, such as phosphorus (P), potassium (K), calcium (Ca), magnesium (Mg), manganese (Mn), zinc (Zn), and copper (Cu) [62].

pH and EC showed the highest positive correlations with Cd, Mn, and Pb and the highest negative correlations with Fe, Cu, Cr, and Ag. As pH increases, the number of negatively charged binding sites on soil particles increases, facilitating the adsorption of these element ions. The observed negative correlations between certain elements and soil properties (pH, EC, and OC) indicate inverse relationships. The absence of soil influence is not always indicated by a negative or weak correlation between trace elements and soil characteristics (pH, EC, and OC). Instead, this pattern is probably linked to the study area’s comparatively narrow, consistently alkaline pH range (7.4–8.6). The impact of soil pH fluctuations on metal solubility and mobility may not be strong enough to yield statistically significant correlations when they occur over a short period [63–65].

In this investigation, OC was positively correlated with Cd, Fe, and Pb, but negatively correlated with Cr and Ni, suggesting that Cd and Pb are mainly bound to organic matter. At the same time, Cr, Cu, and As are retained by iron oxides, clays, and organic matter [66]. The positive relationships among OC, Cu, and Fe may be explained by the role of organic matter in improving soil aeration and reducing nutrient oxidation and precipitation. At the same time, exposure of organic materials to heavy metals can also yield strong metal-binding ligands, such as humic and fulvic substances, through complexation and chelation [67–69]. In addition, most trace elements showed strong interrelationships, especially Ag, indicating possible contamination from common sources; Pb, Cd, and Mn appeared to share one origin, while Ag, Ni, Fe, Zn, Cu, and Cr shared another [68]. Considerable portions of P and Cu were associated with organic matter, whereas Fe, Mn, Zn, Ni, and Cr were mainly linked to sulfide and silicate fractions [70, 71].

In comparison to non-metallophytic agricultural species like *Zea mays* and *Triticum aestivum*, which typically accumulate relatively low levels of trace metals under comparable soil conditions, the concentrations of micro- and potentially hazardous elements in the studied taxa were higher [47, 56, 72]. Certain metals, such as Cr, Cu, S, Mn, Fe, Co, Ni, Zn, and Mo, are micronutrients that naturally occur in small amounts. Still, when present at levels above the allowed limits, they can be toxic and pose a significant hazard to the environment [73]. At low concentrations, trace metals such as Fe, Mn, Zn, Cu, Ni, Cr, and Mo are vital micronutrients for plant metabolism; at higher concentrations, however, they can become hazardous and pose ecological and environmental risks [74]. The weathering of parent materials and human activity can enrich these elements in soils in mining-affected ecosystems, especially in phosphate-mining areas.

The mean concentrations of Mn, Zn, Cu, Pb, Cr, Cd, Ni, and Ag in the soils under investigation were higher than the recommended values for contaminated or agricultural soils. Different regulatory frameworks have different international reference values. For instance, depending on the type of soil and the regulatory requirements, commonly cited guideline ranges include roughly Zn (200–1000 mg/kg), Cu (100–300 mg/kg), Pb (50–500 mg/kg), Cr (75–300 mg/kg), Ni (50–150 mg/kg), and Cd (1–10 mg/kg) [75]. These comparisons suggest that, rather than representing a general exceedance of predetermined “safe limits,” the investigated soils may exhibit increased metal enrichment associated with mining operations.

Plant tissues of the studied species showed elevated levels of Fe, Mn, Zn, Cu, Pb, Cr, Cd, Ni, and Ag, suggesting their ability to tolerate and accumulate metals under contaminated conditions. Since metal reference values in plants vary with species, tissue type, environment, and methods, these concentrations should be viewed as indicators of metal exposure and potential accumulation rather than definitive proof of phytotoxicity [76]. Overall, the results confirm that phosphate mining has influenced metal distribution in both soils and plants, while differences among species highlight variations in metal uptake, tolerance, and phytoremediation potential.

The PCA-biplot ordination of the studied species showed differences in element accumulation among species and within tissues within each species. Metal accumulation in plant tissues is controlled by soil metal availability, species, organ uptake, and translocation processes [77–79]. In addition to the concentration of these elements in the soil, the absorption and translocation of metal ions by plants are influenced by metal plant affinity, plant species, and metal speciation [23, 24].

In the current study, *S. sesban* had the highest potassium content in both shoots and roots, and the biplot confirmed that K was the most effective vector, separating into a distinct group. Among plants that can both accumulate and tolerate high selenium levels, the Fabaceae genus Astragalus stands out as a hyperaccumulator [80]. Hyperaccumulators possess endogenous Se contents ten to one hundred times greater than those of nonaccumulators, as well as a greater S: Se ratio [81]. *Astragalus bisulcatus* is a Se hyperaccumulator [82]. Since Se is a component used in phosphorus mining, as shown in the biplot, *Astragalus vogelii* is the most common Se accumulator among the studied species; however, this finding is limited and is consistent with previous findings by Favorito et al. [83]. Cadmium [84], lead [85], and copper [86] are all associated with *Sesbania drummondi.* Because of their effective antioxidant defense systems, *Sesbania* plants can withstand mercury stress [84]. When *Sesbania cannabina* is grown on diverse fly ash (FA) amendments, it shows significant accumulation of Pb, Mn, Fe, Zn, Cu, and Ni [87]. Most of the elements investigated in this study can be accumulated and stabilized by *S. sesban*, in addition to Cr and Mo, which are more readily transferred to shoot systems. *Senna italica* is a plant that can be used to enhance Cd phytoextraction [88]. Compared with other investigated taxa, *Senna italica* is regarded as a hyperaccumulator of Cd and Cr in the manufacturing environment of Saudi Arabia, according to a study by Towanou et al. [89]. According to Badr et al. [90]., *Senna italica* was influential in the accumulation of Co, Pb and Cu. Our findings also indicate that *Senna italica* may help accumulate the microelements under study.

In a biplot, *Trigonella hamosa* has been shown to accumulate numerous trace elements associated with phosphate in its shoots and roots, and *Trigonella foenum-graecum* can be used for phytoremediation of Cd [91]. According to Steliga and Kluk, *Melilotus officinalis* is suitable for the phytoremediation of petroleum hydrocarbon-polluted soil and for the removal of zinc, lead, and mercury [92]. Furthermore, the potential for the phytoextraction of Mo- and Cu-polluted soils by *Melilotus officinalis* was estimated by Ghazaryan et al. [93]. According to Naz et al. [94]., *Melilotus indicus* successfully phytostabilized Cd in the Pakistani Hayat Abad Industrial Estate; the findings of the present study are consistent with these. The accumulation characteristics of *Bidens pilosa*, a possible Cd hyperaccumulator, were identified by Sun et al.. and Dai et al. [95, 96]. The *Bidens pilosa* Pb accumulation profile reported by Salazar et al. [97]. is consistent with our data. According to field tests, *Conyza sumatrensis* significantly reduces Cd levels [98]. These results, along with the latest information, bolster the notion that *C. bonariensis* plants might be effective for the phytoremediation of harmful trace elements (TEs) in contaminated areas [6].

In the present study, most metals had BF and BAC values greater than unity, but the TF values were less than unity because several metals were concentrated in the roots. For every taxon under study, the TF values for Mn, Se, Cu, Cd, B, Ni, Si, Na, and Fe (apart from *Astragalus*, TF > 1) and Zn (apart from *Melilotus*, TF > 1) were less than one. These findings are consistent with several earlier studies [25, 48, 99, 100]. Plants with low translocation factors (TFs) and high BF are potential candidates for phytostabilization. Most of the species under investigation demonstrated potential for phytostabilization and phytoextraction, as indicated by the recorded BAC, BF, and TF values.

Bioaccumulation factor (BF) and translocation factor (TF) are used to estimate the phytoremediation potentials of plants [101–103]. According to [101, 104], species with BFs value > 1 and TFs < 1 might be helpful for phytostabilization purposes, while species with BFs = TFs > 1 might be suitable for phytoextraction purposes. Although some species exhibited BAC and TF values greater than unity for specific metals, the measured shoot concentrations did not exceed the widely accepted thresholds for classifying plants as hyperaccumulators. Hyperaccumulator plants are typically defined by element-specific shoot concentrations exceeding established criteria (e.g., > 1000 mg/kg for Ni, Cu, Co, and Pb; >10,000 mg/kg for Zn and Mn; and > 100 mg/kg for Cd) [102]. Therefore, the studied species are better described as exhibiting metal accumulation potential rather than actual hyperaccumulator characteristics.

Species with low TF values and relatively high BF may show a tendency toward metal retention in roots, suggesting phytostabilization [105]. However, the application of these species for field remediation needs to be considered in light of various factors. For example, the long-term stability of immobilized metals, remobilization of metals under changing soil conditions, erosion processes, and management of plant biomass. In addition, the possibility of metal transfer through ecological processes, such as herbivory or food chain transfer, needs to be assessed before large-scale application. Thus, the findings of the present study should be considered a preliminary assessment of the phytostabilization potential rather than a direct application in field remediation. Further studies on biomass production, long-term metal stability, and ecological safety are recommended to confirm the suitability of these species for phytoremediation. Plant species accumulate different elements simultaneously, so the element accumulation index (EAI) was used to assess the overall performance of macronutrient accumulation in the plant species studied. In terms of the element accumulation index (EAI) (Fig. 5), *T. hamosa* and *C. bonariensis* shoots had the highest EAI values. Thus, these species are better able to accumulate macronutrients and are therefore more suitable for phytoextraction purposes.

Our study indicates that indigenous plant species near the Abu-Tartur mining site can be effective for phytoremediation, highlighting their significance for land rehabilitation in metal-contaminated areas. Identifying resilient species is crucial for restoring vegetation. Notably, five of the studied species are from the Fabaceae family, known for traits such as nitrogen fixation, which can impact metal mobility. However, this focus on specific species limits the analysis of differences in metal uptake. Future research should include non-mining reference sites and diverse conditions to more comprehensively evaluate plant adaptation and metal accumulation patterns.

## Conclusion

This study provides a preliminary assessment of metal accumulation patterns and phytoremediation potential in native plant species growing near the Abu-Tartur mining site. The results revealed interspecific differences in metal uptake, retention, and translocation, suggesting that some species may have potential for phytostabilization or phytoextraction under the studied conditions. However, these findings should be interpreted with caution, as the investigated species showed metal accumulation potential but did not exhibit confirmed hyperaccumulator characteristics. In addition, given the limitations in spatial coverage and sampling design, further research is required to evaluate biomass production, long-term metal stability, ecological safety, and performance under a wider range of environmental conditions before any practical field application can be recommended.

## Supplementary Information

Supplementary Material 1.

## Data Availability

All recorded, measured, and analyzed datasets generated during this study are included in the main text of the manuscript. The reported data are based on ten biological replicates per species (*n* = 10). Where applicable, technical replicate measurements were averaged within each biological replicate before statistical analysis.
