# Supplementary material for: Soil–plant interactions and metal uptake efficiency of native species in phosphate mining-affected environments
Source: BMC Plant Biol. 2026 May 11;26:829. doi: 10.1186/s12870-026-08832-z (PMC13159367; doi:10.1186/s12870-026-08832-z)
Supplement: Supplementary file 1 — Supplementary Material 1. [file 12870_2026_8832_MOESM1_ESM.docx]

**Table S1. Estimated limits of detection (LOD) for analyzed elements using ICP-AES under the applied analytical conditions.**

| **Element** | **LOD (mg kg⁻¹)** |
| --- | --- |
| Fe | 0.5 |
| Mn | 0.1 |
| Zn | 0.05 |
| Cu | 0.05 |
| Pb | 0.2 |
| Cd | 0.01 |
| Ni | 0.1 |
| Cr | 0.1 |
| Mo | 0.05 |
| Se | 0.1 |
| B | 0.1 |
| Ag | 0.02 |
| Si | 0.5 |
| Na | 0.5 |
